# Meta-analysis of the clinical value of abnormally expressed long non-coding RNAs for pancreatic cancer

**DOI:** 10.18632/oncotarget.20803

**Published:** 2017-09-11

**Authors:** Liangliang Lei, Jianguang Wang, Like Zhang, Yanbin Chen, Pengfei Yuan, Dechun Liu

**Affiliations:** ^1^ Department of Gastrointestinal Surgery, The First Affiliated Hospital, and College of Clinical Medicine of Henan University of Science and Technology, Luoyang, Henan, China

**Keywords:** pancreatic cancer, lncRNA, diagnosis, prognosis, meta-analysis

## Abstract

Pancreatic cancer (PC) is one of the most lethal malignant neoplasms of the digestive system. Long non-coding RNAs (lncRNAs) are a novel type of non-protein coding transcripts that play an important role in pancreatic carcinogenesis. We herein aimed to meta-analyze the diagnostic and prognostic significance of lncRNA expression profiles in PC. A comprehensive retrieval of eligible studies was performed based on the online databases. Quantitative meta-analyses of the pooled diagnostic parameters and hazard ratios (HRs) were enabled by using standard statistical methods. A total of 16 studies comprising 1386 PC patients were included. The pooled effect sizes exhibited that lncRNA expression profile achieved a combined sensitivity of 0.82 (95% CI: 0.72–0.89), specificity of 0.77 (95% CI: 0.65–0.86) and AUC (area under curve) of 0.87 (95% CI: 0.83–0.89) in distinguishing patients with PC from noncancerous controls. Notably, abnormally expressed lncRNAs were markedly associated with unfavorable overall survival (OS) in PC (univariate analysis: HR = 1.52, 95% CI: 1.04–2.22, *P =* 0.031; multivariate analysis: HR = 1.55, 95% CI: 1.19–2.02, *P =* 0.001). Statistical significance was also observed in our stratified analyses grouped by clinicopathologic features. In conclusion, abnormal lncRNA expression profiles could be rated as promising biomarker(s) to enable diagnosis and predict the prognosis of PC.

## INTRODUCTION

Pancreatic cancer (PC) remains one of the most common malignancies of the digestive system, accounting for the major causes of cancer-related death worldwide [1]. In China, although PC caused deaths in male only ranks the 6th of the cancer mortality rates [2], it features a very unfavorable prognosis. The diagnostic potentials of current imaging technologies as well as the conventional serum biomarkers for PC are limited due to the restricted sensitivity and specificity [3]. Therefore, it is imperative to develop novel useful biomarker(s) to help diagnosis and facilitate prediction of the clinical outcomes in PC.

The “long noncoding RNAs” (lncRNAs) are a group of noncoding RNAs with the sequences of 200 bp to 10 kb in length, but have no functional protein-coding frame(s) [4]. LncRNAs are now known to represent important players in evolutionary and developmental biology of the vertebrates [5, 6]. The deregulation of lncRNAs is implicated in multiple human malignancies, including PC [7, 8]. As reported, many types of lncRNAs are shown to be involved in the pathogenesis and progression of PC, such as UCA1 [9, 10], AFAP1-AS1 [10], HOTAIR [11], and MALAT-1 [12, 13], and so forth. Of note, studies have shone a spotlight on a set of lncRNAs that could be popularized as diagnostic or prognostic biomarkers for PC [9–24]. For example, the reported HOTAIR and PVT1 lncRNA set could distinguish PC patients from cancer-free individuals with sensitivities and specificities ranging from 60% to 97%, showing a large potential to be novel non-invasive indicator(s) to aid in PC diagnosis [24]. Importantly, the prognostic significance of single lncRNA expression profile in PC has been extensively studied and highlighted as well [9–11, 13–23]. However, there are often large heterogeneity and bias across single studies owing to limited sample sizes and study design, which may finally compromise the study accuracies even conclusions. Based on published evidence, we herein undertake a comprehensive meta-analysis according to standard methods, with the purpose of giving an overview of the clinical utilities of lncRNA expression profiles as novel non-invasive biomarkers for PC.

## RESULTS

### Search results and study characteristics

**Figure 1:**
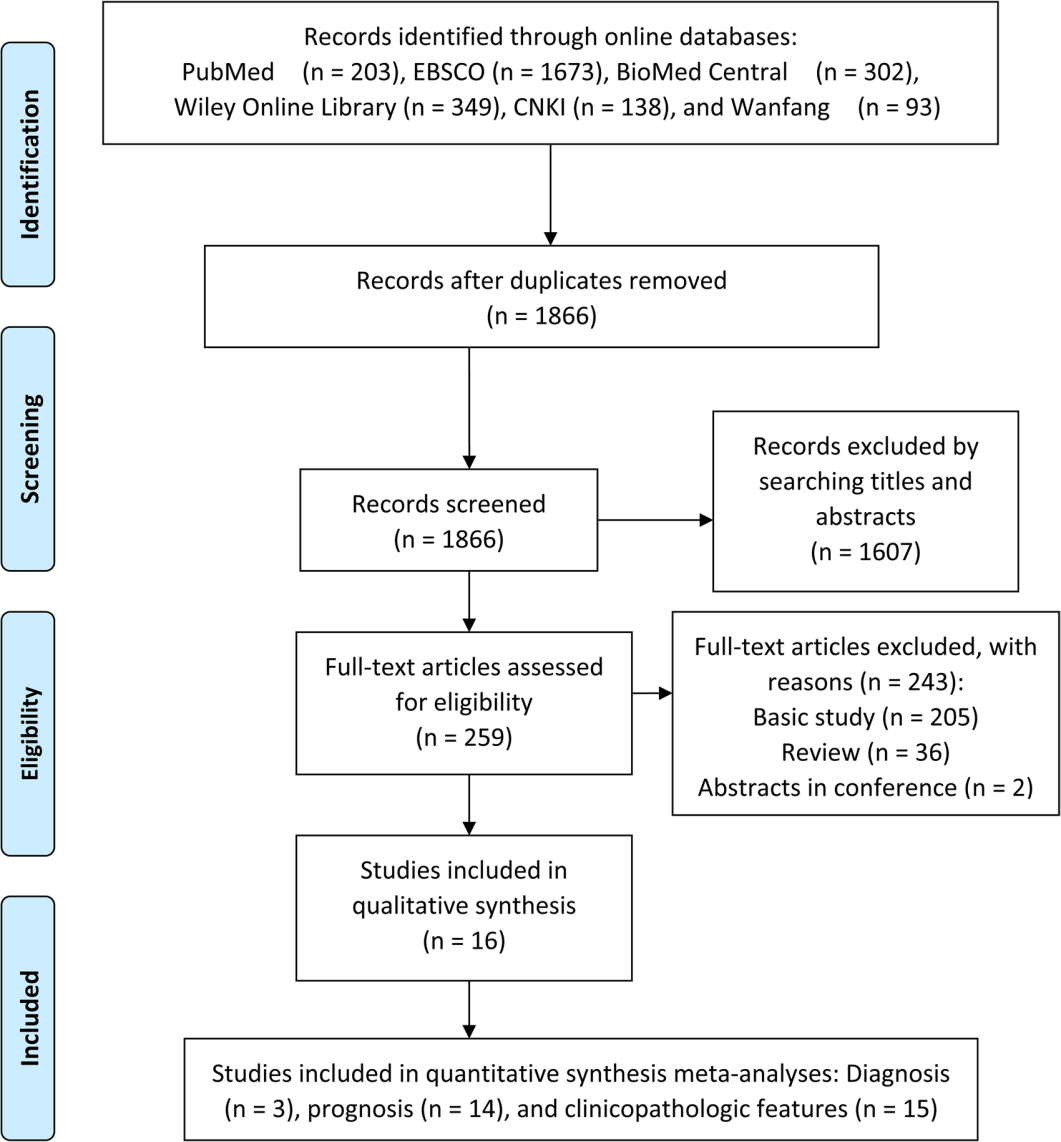
Study enrollment procedure in terms of the standards of the PRISMA diagram

Studies were included and excluded in line with the standards of the PRISMA diagram (Figure 1). In the identification set, a total of 1866 records were obtained from the online databases after removing the duplicates. In the screening set, two authors independently judged the records by reading titles and abstracts, and 1607 studies uncorrelated to our topic were excluded. The left 259 studies received full-text evaluation for eligibility, and 36 of them were identified as reviews, 205 were basic studies, and therefore were all discarded. Finally, a total of 16 studies involved 3 for diagnosis [12, 15, 24], 14 for prognosis [9–11, 13–23], and 15 for clinicopathologic features [9–23], were included in the qualitative synthesis.

The studies comprised a pooled patient size of 1386, including 247 cases for the diagnostic synthesis, and 1139 cases with survival data for the prognostic meta-analysis. In addition, 177 noncancerous controls were included in the diagnostic synthesis. The control entities involved normal pancreatic tissues [15, 17, 24], matched adjacent non-tumor pancreatic tissues [9–14, 20, 22, 23], benign pancreatic lesions [24], and adjacent normal tissues [16, 18, 19, 21]. The diagnoses of PC were all confirmed histopathologically, and all tissue samples were obtained from patients undergoing resection surgery prior to other therapies. The primary endpoints included OS [9–11, 13–23] and DSS [12], which were all statistical analyzed using the Kaplan-Meier analysis and log-rank test. Expression level of the lncRNAs was measured by the approach of quantitative real-time polymerase chain reaction (qRT-PCR), and the reference genes utilized for endogenous normalization covered GAPDH [9–11, 17, 18, 22, 23], RNU6B [14, 21] and β-actin [13, 16, 20, 24]. We also included one study conducted based on the GEO database [15] (Table 1).

**Table 1:** Main features of all included studies for diagnosis and prognosis

Author	Year	Country	Patient size	Sample type	Control type/number	LncRNA signature	Method	Reference gene	Survival point	Follow-up time	NOS score	QUADAS score	References
Chen et al.	2016	China	128	tissue	non-tumor tissue/128	UCA1	qRT-PCR	GAPDH	OS	60	6	NA	9
Kim et al.	2013	America	102	tissue	noncancerous tissue/102	HOTAIR	qRT-PCR	GAPDH	OS	Unclear	8	NA	11
Fu et al.	2016	China	80	tissue	non-tumor tissue/80	CRNDE, NR_036488, ENSG00000244649, AFAP1-AS1, UCA1, ENSG00000218510	qRT-PCR	GAPDH	OS	46	7	NA	10
Wei et al.	2017	China	64	tissue	adjacent normal tissue/64	XIST	qRT-PCR	RNU6B	OS	Unclear	6	NA	21
Peng et al.	2016	China	40	tissue	adjacent normal tissue/40	CCDC26	qRT-PCR	GAPDH	OS	60	8	NA	18
Sun et al.	2016	China	150	tissue	adjacent normal tissue/150	HMlincRNA717	qRT-PCR	U6	OS	60	7	NA	19
Liu et al.	2016	China	103	tissue	adjacent non-tumor tissue/103	uc.345	qRT-PCR	RNU6B	OS	> 40	6	NA	14
Pang et al.	2014	China	126	tissue	adjacent non-tumor tissue/126	MALAT1	qRT-PCR	β-actin	OS	60	7	NA	13
Li et al.	2015	China	90	tissue	normal adjacent tissues/90	Linc00675	qRT-PCR	β-actin	OS	60	7	NA	16
Zheng et al.	2016	China	106	tissue	noncancerous tissue/106	LOC389641	qRT-PCR	GAPDH	OS	> 84	8	NA	23
Ding et al.	2014	China	85	tissue	adjacent non-tumor tissue/85	LOC285194	qRT-PCR	GAPDH	OS	median 10.2	7	NA	22
Sun et al.	2014	China	35	tissue	adjacent noncancerous tissue/35	ENST00000480739	qRT-PCR	β-actin	OS	< 30	6	NA	20
Li et al.	2014	China	30	tissue	normal tissue/30	BC008363	qRT-PCR	GAPDH	OS	median 15	6	NA	17
Liu et al.	2014	China	45	tissue	adjacent noncancerous tissue/45	MALAT1	qRT-PCR	GAPDH	DSS	< 40	6	9	12
Xie et al.	2016	China	55	Saliva	healthy control/55	HOTAIR, PVT1	qRT-PCR	β-actin	NA	NA	NA	10	24
Xiong et al.	2017	GEO database	147	tissue	normal tissue/77	NEAT1	qRT-PCR	Unclear	NA	NA	NA	NA	15

OS: Overall survival; DSS: disease-specific survival; QUADAS: Quality Assessment for Studies of Diagnostic Accuracy; NOS: Newcastle-Ottawa Scale; qRT-PCR: quantitative real-time polymerase chain reaction; NA: not applicable.

### Study quality and heterogeneity

Study quality judged by the 14-item QUADAS checklist revealed that no studies were in accordance with one of the item “blinding of researchers to index test” (Supplementary Table 1). However, all the diagnostic studies obtained a cumulative evaluation score larger than 8 (Supplementary Table 1); likewise, the NOS checklist also showed an evaluation score equal or greater than 6 for each retrospective cohort study, indicating that the risk of bias across studies was relatively small (Supplementary Table 2).

Heterogeneity analysis showed that there was large degree of heterogeneity existed in the pooled diagnostic studies (Q = 9.955, df = 2.00, *P* = 0.003, *I*^2^ = 79.9%) as well as in the prognostic studies by univariate analysis (*Chi*^2^ = 70.33, df = 14.0, *P* < 0.001, *I*^2^ = 80.1%) and multivariate analysis (*Chi*^2^ = 49.84, df = 154.0, *P* < 0.001, *I*^2^ = 69.9%). Similarly, heterogeneity was also generated in our stratified analyses within the subgroup of tumor size, depth of invision, tumor stage, lymphatic metastasis and distant metastasis (the estimated *I*^2^ are indicted Table 2). Heterogeneity analyses of the pooled studies by visual L’Abbe and Galbraith plots are shown in Supplementary Figure 1.

**Table 2:** Summary of the subgroup analyses of the association between OS and clinicopathological features in PC

Variables	Univariate analysis					Multivariate analysis				
	Included studies	HR (95% CI)	*P* value	I^2^ (%)	Effect model	Included studies	HR (95% CI)	*P* value	I^2^ (%)	Effect model
Age	12	1.01 (0.99–1.03)	0.387	17.6	Fixed					
Gender	12	1.05 (0.89–1.23)	0.588	11.6	Fixed					
Location	4	1.18 (0.90–1.55)	0.233	3.6	Fixed					
Histological grade	9	1.27 (1.07–1.50)	0.006	0	Fixed					
Tumor size	11	1.20 (0.82–1.74)	0.353	76.7	Random	7	1.48 (1.03–2.13)	0.036	58.6	Random
Depth of invision	7	1.33 (0.91–.94)	0.137	60.6	Random	4	1.19 (0.77–1.83)	0.443	63.5	Random
Tumor stage	10	1.211 (0.79–1.86)	0.381	83	Random	9	1.32 (0.82–2.12)	0.247	80.7	Random
Lymphatic metastasis	11	1.42 (1.03–1.94)	0.03	70.5	Random	7	1.55 (1.03–2.35)	0.038	52.2	Random
Nervous invasion	6	1.12 (0.89–1.41)	0.326	43.2	Fixed	2	1.22 (0.28–5.26)	0.791	89.7	Random
Venous invasion	4	1.16 (0.84–1.62)	0.366	0	Fixed					
Distant metastasis	4	1.64 (0.86–3.12)	0.131	73.1	Random					

### Diagnostic performance

**Figure 2:**
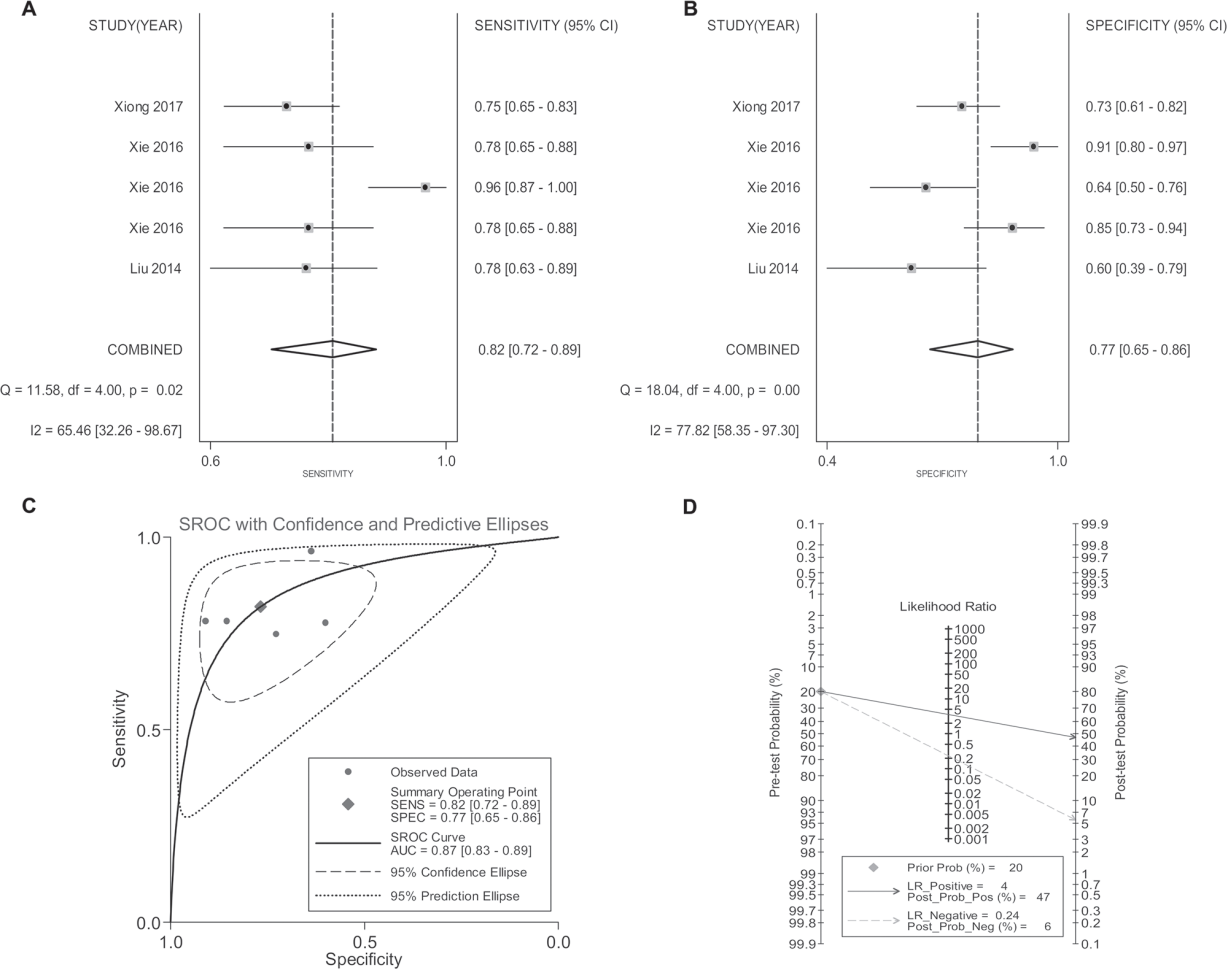
Forest plots of pooled sensitivity (**A**), specificity (**B**), SROC curve (**C**) and Fagan’s plot (**D**) for the overall combined diagnostic effect size.

For the ability of discriminating patients with PC from noncancerous individuals, lncRNA expression profile exhibited a pooled sensitivity of 0.82 (95% CI: 0.72–0.89), specificity of 0.77 (95% CI: 0.65–0.86), PLR of 3.52 (95% CI: 2.30–5.39), NLR of 0.24 (95% CI: 0.15–0.36), DOR of 14.96 (95% CI: 7.94–28.17) and AUC of 0.87 (95% CI: 0.83–0.89). Fagan’s plot analysis of lncRNA signature testing showed an increase of pos*t*-test probability (at 20%) of positive result to 47% and a decrease of the negative result to 4%. The forest plots of pooled sensitivity, specificity, SROC curve as well as the Fagan’s plot are displayed in Figure 2.

### Prognostic significance

**Figure 3:**
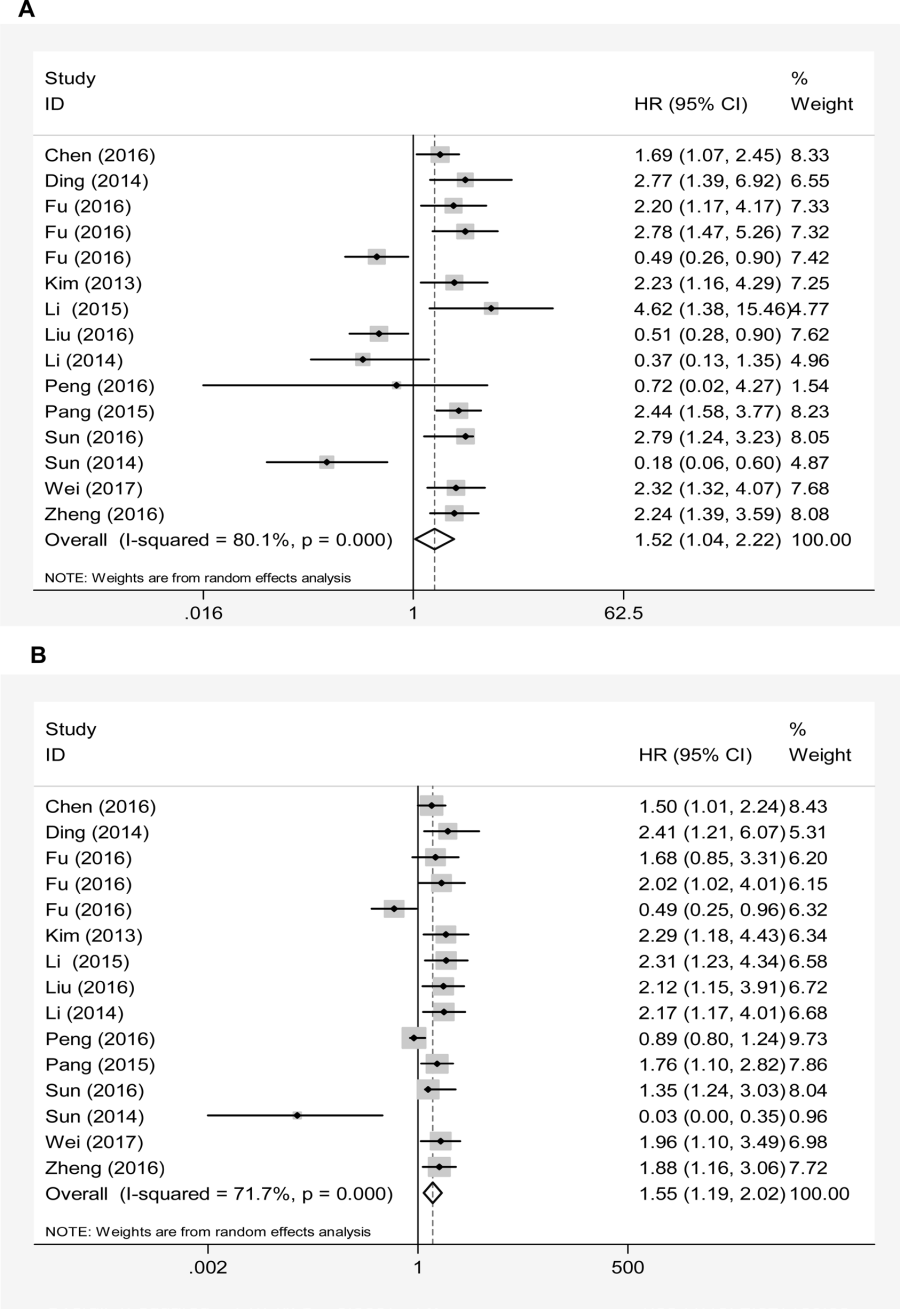
Forest plots of pooled HRs with 95% CIs for the overall combined prognostic meta-analysis by (**A**) univariate analysi and (**B**) multivariate analysis.

A significant association between abnormally expressed lncRNAs and poor overall survival (OS) of PC patients was observed in both of the univariate analysis (HR = 1.52, 95% CI: 1.04–2.22, *P* = 0.031, Figure 3A) and multivariate analysis (HR = 1.55, 95% CI: 1.19–2.02, *P* = 0.001, Figure 3B), suggesting that lncRNA-based molecular testing retains a promising predictive value in monitoring the prognosis of PC.

### Influence analysis and subgroup study

**Figure 4:**
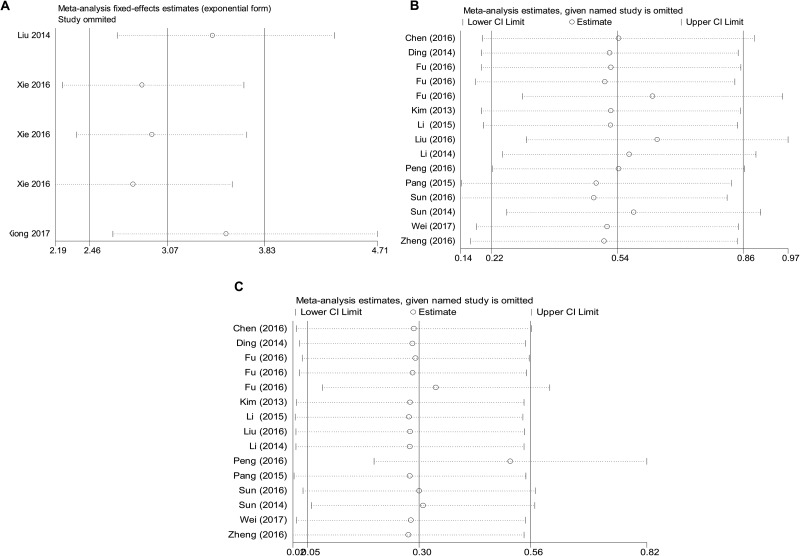
Sensitivity analysis of the overall combined diagnostic meta-analysis (**A**) and prognostic meta-analysis (**B)** for univariate analysis; (**C**) for multivariate analysis)

Influence analysis was employed to trace the outlier values across combined effect sizes. As exemplified in Figure 4, the fixed effect estimates displayed no outlier values either in the diagnostic meta-analyses or the prognostic studies, indicating that our results were relatively reliable.

Further stratified study grouped by clinicopathologic features, the results exhibited that the prognosis (OS) of patients with PC was markedly associated with histological grade (univariate analysis: HR = 1.27, 95% CI: 1.07–1.50, *P* = 0.006), tumor size (multivariate analysis: HR = 1.48, 95% CI: 1.03–2.13, *P* = 0.036), and lymphatic metastasis (univariate analysis: HR = 1.42, 95% CI: 1.03–1.94, P = 0.030; multivariate analysis: HR = 1.55, 95% CI: 1.03–2.35, *P* = 0.038) (Table 2). Other clinicopathologic factors included age, gender, location, depth of invision, tumor stage, nervous invasion, venous invasion, and distant metastasis showed no significance to the prognosis of PC (Table 2). For the relationship between lncRNA expression and clinicopathological characteristics, the data demonstrated that tumor size (combined *P* < 0.001), depth of invision (combined *P* = 0.014), tumor stage (combined *P* < 0.001), lymphatic metastasis (combined *P* = 0.001) and distant metastasis (combined *P* < 0.001) were significantly correlated to lncRNA expression levels in PC (Table 3).

**Table 3:** The associations between lncRNA expression and clinicopathological factors in PC

Studies	LncRNA signature	LncRNA expression and clinicopathological factors (*P* value)										
		Age	Gender	Location	Histological grade	Tumor size	Depth of invision	Tumor stage	Lymphatic metastasis	Nervous invasion	Venous invasion	Distant metastasis
Chen 2016 [9]	UCA1	0.321	0.585	0.457	0.156	0.021	0.033	0.013	0.073	0.092	0.102	/
Fu 2016 [10]	CRNDE	0.116	0.822	0.478	0.501	0.501	/	0.37	0.651	0.502	/	1
Fu 2016 [10]	NR_036488	0.262	0.822	0.237	1	0.262	/	0.37	0.366	0.823	/	0.456
Fu 2016 [10]	ENSG00000244649	0.501	0.26	0.813	0.116	0.501	/	0.37	0.651	0.263	/	1
Fu 2016 [10]	AFAP1-AS1	0.116	0.26	0.813	0.823	0.044	/	0.654	0.651	0.502	/	0.456
Fu 2016 [10]	UCA1	0.262	0.26	0.813	0.262	0.823	/	1	1	0.823	/	1
Fu 2016 [10]	ENSG00000218510	0.501	0.499	0.478	0.044	0.044	/	0.654	0.366	0.502	/	0.005
Wei 2017 [21]	XIST	0.798	0.317	/	/	0.006	/	0.023	0.131	0.127	/	0.079
Peng 2016 [18]	CCDC26	0.341	0.748	/	0.105	0.022	/	/	0.2	/	0.205	/
Sun 2016 [19]	HMlincRNA717	0.5	0.21	/	0.325	0.001	/	0.001	0.003	/	/	0.001
Liu 2016 [14]	uc.345	0.426	0.304	0.183	/	0.549	0.01	0.031	0.33	/	/	/
Liu 2014 [12]	MALAT1	0.259	0.989	0.321	0.334	0.019	0.025	0.004	0.369	0.553	0.954	0.103
Pang 2014 [13]	MALAT1	0.591	0.371	/	0.216	0.001	/	0.001	0.001	/	/	0.001
Li 2015 [16]	Linc00675	0.833	0.512	/	0.304	/	0.697	/	/	0.006	/	/
Zheng 2016 [23]	LOC389641	0.435	0.558	/	0.529	/	0.495	0.024	0.006	0.12	/	/
Ding 2014 [22]	LOC285194	0.536	0.124	/	0.306	0.976	0.625	0	0	/	/	/
Sun 2014 [20]	ENST00000480739	0.23	0.404	/	0.378	0.564	/	0.035	0	0.432	/	/
Li 2014 [17]	BC008363	0.721	0.785	/	/	0.554	0.47	0.346	0.714	0.721	0.242	/
Pooled *P* value		0.5	0.719	0.709	0.132	< 0.001	0.014	0	0.001	0.123	0.221	< 0.001

### Publication bias

**Figure 5:**
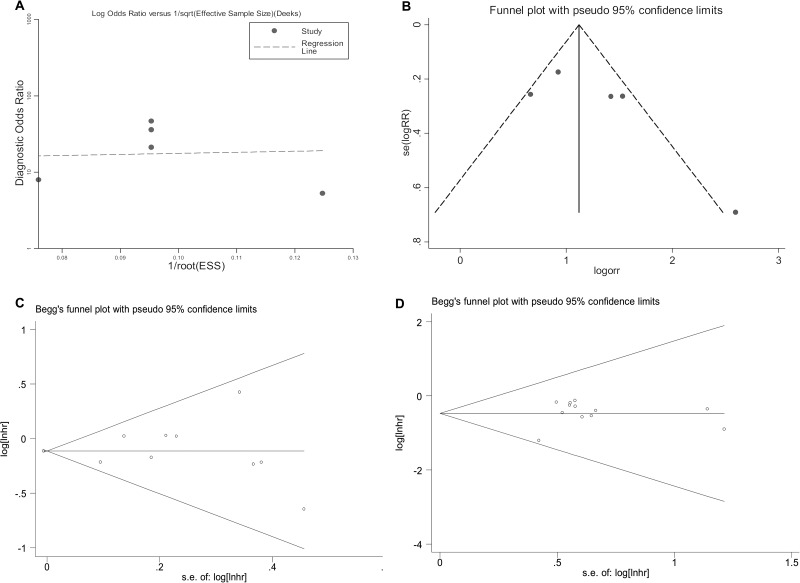
Publication bias assessed by Deek’s funnel plot asymmetry test (**A**) and Funnel plot analysis (**B**) in diagnostic meta-analysis; and by Begg’s funnel test in the pooled prognostic studies (**C**) for univariate analysis; (**D**) for multivariate analysis).

Deek’s funnel plot asymmetry test showed an estimated *P* value of 0.927 for the diagnostic studies (Figure 5A). Correspondingly, the Funnel plot analysis also displayed no obvious asymmetry among the overall studies (Figure 5B). Besides that, the Egger and Bgger tests, as well as the Funnel plot analysis all showed no evidence of statistically significant publication bias across the overall combined prognostic studies, with P>0.05 (Figure 5C, 5D and Supplementary Figure 2)

## DISCUSSION

Currently, the major difficulty in treating pancreatic cancer (PC) is the late onset of symptoms. Patients with PC often undergo worse clinical outcomes and the 5-year survival rate was estimated lower than 25% [25]. There are now promising data that have highlighted the potential of abnormally expressed lncRNAs as novel biomarkers to inform the clinical management of PC [9–24]. In order to provide valid evidences, we undertook a meta-analysis of 16 studies comprising 1386 PC patients and assessed the clinical utilities of lncRNA expression profiles as biomarkers for PC diagnosis and prognosis.

The diagnostic significance of molecular based lncRNA profiling in digestive system tumors has been documented by the recent studies [26, 27]. In the first part of our analysis, we included studies that evaluated the diagnostic performance of abnormally expressed lncRNA(s) for PC. Our pooled effect sizes for diagnosis reveled that lncRNA signature harbored a sensitivity of 0.82, specificity of 0.77 and AUC of 0.87 in differentiating patients with PC from noncancerous controls. Moreover, the estimated PLR suggested that the true judgment of positive results in lncRNA testing yields a ratio of 3.52 over the false judgment. Yet, the pooled NLR of 0.24 also indicated that the false judgment of negative results in lncRNA testing retains a ratio of 0.24 over the true judgment. Importantly, the pooled DOR was shown to be 14.96, which is larger than 1.0, also revealed a powerful capacity of lncRNA signature for PC diagnosis. These data mentioned above suggested lncRNA expression signature confers a relatively high diagnostic efficacy in the management of PC, and therefore could be developed as additional biomarker(s) to aid in PC diagnosis.

We further evaluated the efficacy of lncRNA expression profile as an independent marker for PC prognosis. Through systematic analysis, we found that altered expression of lncRNA profile was significantly associated with poor overall survival (OS) time of PC (HR = 1.52, with 95% CI: 1.04–2.22, and *P* = 0.031 in univariate analysis; HR = 1.55, with 95% CI: 1.19–2.02, and P = 0.001 in multivariate analysis). Correspondingly, a published meta-analysis has documented that lncRNA expression profile predicted worse clinical outcomes in forecasting prognosis of osteosarcoma. [28]. Other available evidence from Cui et al also supported our results [29].

For the relationship between clinicopathological characteristics and lncRNA expression, our stratified analysis evidenced that the clinicopathological factors as tumor size, depth of invision, tumor stage, lymphatic metastasis and distant metastasis were markedly associated with lncRNA expression. Moreover, the prognostic implication of clinicopathological factors on the OS of PC was also showed in our stratified analyses based on histological grade, tumor size, and lymphatic metastasis. However, unlike the conclusions of the pooled analyses, some single studies revealed no significant correlations between lncRNA expression and tumor size [10, 14], depth of invision [16, 17, 22, 23], tumor stage [10, 17], lymphatic metastasis [10] or distant metastasis [10]. As the lncRNA testing profiles were differed among studies, we speculate that different lncRNAs may exert diverse biological functions in PC and resulted in different clinical outcomes. Thus, more evidences are required to further testify this conclusion.

On the other hand, we observed large degree of heterogeneities among included studies and we also try to well interpret the causes from different aspects [30]. For the lncRNA expression signature, a total of 16 kinds of lncRNAs were evaluated and resulted in different expression status. Besides that, different studies utilized non-unified reference gene in determining the validity of the results. For the diagnostic meta-analysis, the small sample size may contribute to the study bias although on obvious publication bias was observed. All these factors may contribute to the causes of heterogeneities across studies. Nevertheless, our sensitive analysis identified no outlier studies, hinting that our results were relatively reliable.

Our study still has several limitations: (1) The sample sizes of the diagnostic meta-analysis are small and the clinical relevance of our findings are limited; (2) Study heterogeneity in some of our analyses was large; (3) The HRs and 95% CIs from 2 articles could not be directly obtained and were estimated by software, which may declined the overall accuracy of the pooled effects. Consequently, the conclusions form our study could not fully mirror the real clinical significance of lncRNA signature in PC and should therefore be confirmed by other larger sample, multicenter and randomized controlled prospective studies.

In summary, data from the current study may help to understand the significance of lncRNA expression signature on the diagnosis and prognosis of PC. Abnormal lncRNA expression profiling may serve as a novel biomarker to aid in diagnosis and predict the prognosis of PC. The suitable single or parallel lncRNA expression pattern(s) with high efficacies for diagnosis and/or prognosis should be identified in future.

## MATERIALS AND METHODS

### Search strategies

This meta-analysis was performed and reported in accordance with the criteria issued in the Preferred Reporting Items for Systematic Reviews and Meta-Analyses statement [31]. We searched the online PubMed, EBSCO, Wiley Online Library, BioMed Central, CNKI, and Wanfang databases for retrieval of eligible studies up to April 30, 2017. The utilized search items were (“pancreatic cancer” or “pancreatic carcinoma” or “carcinoma of pancreas” or “pancreatic tumor”) and (“long non coding RNA” or “lncRNA” or ““non coding RNA”) and (“prognosis” or “outcome” or “survival” or “hazard ratio” or “HR” or “follow-up” or “predict” or “diagnosis” or “sensitivity” or “specificity” or “area under the curve” or “AUC”). We also conducted manual searching for eligible studies from article references.

### Selection criteria

Studies were firstly judged by reading the titles and abstracts, and was included if they fulfilled our topic. All of the initially enrolled studies received full-text evaluation and were finally included for the meta-analyses if they fitting the following criteria: (1) the PC patients had a definite diagnosis by pathologic examinations and the paired controls were from noncancerous tissues or cancer-free individuals; (2) studies evaluated the diagnostic and/or prognostic significance of lncRNA signature (single or in parallel) in PC; (3) either the estimated sensitivity, specificity or AUC were available in the diagnostic studies, or the HR with 95% CI for OS (overall survival), PSF (progression free survival), DFS (disease-free survival) or DSS (disease-specific survival) were clear in prognostic studies; (4) studies were published in English or Chinese and had sample numbers larger than 20. Studies not fitting the above inclusion criteria as well as the status of review articles, basic research, animal studies, comments, letters or conference abstracts all would be excluded.

### Data extraction

For each included study, data were extracted in duplicate by two reviewers. The following information were retrieved: study design, author names, published date, country/ethnicity, sample size/type, sensitivity, specificity, AUC, follow-up time, HR with 95% CI for OS, PSF, DFS, DSS and clinicopathologic features (age, gender, location, histological grade, tumor size, depth of invision, tumor stage, lymphatic metastasis, nervous invasion, venous invasion and distant metastasis), etc. Any disagreements were resolved by group discussion.

### Quality and bias assessment

Quality and bias assessment were conducted by two reviewers independently. For the diagnostic studies, the Quality Assessment for Studies of Diagnostic Accuracy (QUADAS) checklist contains 14 criteria was employed [32], wherein, the risk of bias was assessed as “low”, “high” or “unclear” for each criteria. Study received a “low” risk judgment will get a score of “1”. Either a judgment evaluated as “high risk” or “unclear” will be scored as “0”. If a cumulative score is higher than 8, the study could be deemed as low risk of bias. On the other hand, the Newcastle-Ottawa Scale (NOS) checklist was applied as a tool in assessing bias from the retrospective cohort studies [33], in which bias from cohort selection, comparability and outcome ascertainment were evaluated, with a cumulative score of 9. Study has an overall evaluation score greater than 6 was considered to be of high quality.

### Statistical analysis

Effect sizes were combined with the programs of Stata 12.0 (Stata Corporation, USA) according to the standard method. The duplicates among enrolled eligible studies were checked using Endnote X7 software (EndNote Clarivate Analytics, USA). Statistical tests of heterogeneity were using *Chi*^2^ and *I*^2^ tests as well as the L’Abbe and Galbraith plot analysis. The pooled effect sizes were deemed as heterogeneous either *P* < 0.01 for *Chi*^2^ test or *I*^2^ > 50% for *I*^2^ test. A fixed-effect model will be chosen for aggregation of the pooled results if no heterogeneity appeared among studies, otherwise, a random-effect mode will be selected. In the diagnostic meta-analysis, the pooled sensitivity, specificity, positive likelihood ratio (PLR), negative likelihood ratio (NLR), diagnostic odds ratio (DOR), and AUC with corresponding 95% CIs were obtained. In the prognostic meta-analysis, the HRs with corresponding 95% CIs were meta-analyzed. Assessment of the bias among publications was enabled by using the Deek’s funnel plot asymmetry test, visual Funnel plot, as well as Egger and Bgger tests, all with a significant level at *P* < 0.05.

## SUPPLEMENTARY MATERIALS FIGURES AND TABLES


