# Supplementary material for: Meta-analysis of the clinical value of abnormally expressed long non-coding RNAs for pancreatic cancer
Source: Oncotarget. 2017 Sep 11;8(51):89149–59. doi: 10.18632/oncotarget.20803 (PMC5687677; doi:10.18632/oncotarget.20803)
Supplement: Supplementary file 1 [file oncotarget-08-89149-s001.pdf]

Meta-analysis of the clinical value of abnormally expressed long non-coding RNAs for pancreatic cancer

SUPPLEMENTARY MATERIALS

Supplementary Table 1: Study bias among the diagnostic studies assessed by the 14-item QUADAS checklist

Study	Item 1	Item 2	Item 3	Item 4	Item 5	Item 6	Item 7	Item 8	Item 9	Item 10	Item 11	Item 12	Item 13	Item 14
Liu 2014	1	1	1	1	1	1	1	1	1	1	0	1	1	U
Xie 2016	U	1	1	1	1	1	1	1	1	1	0	1	1	U
Xiong 2017*	NA	NA	NA	NA	NA	NA	NA	NA	NA	NA	NA	NA	NA	NA

NA: Not applicable. * Data from the study were based on the GEO database.
Note:
Item 1: Representative spectrum ?; Item 2: Acceptable selection criteria ?; Item 3: Acceptable reference standard ?; Item 4: Short time period between tests ?; Item 5: All participants received reference standard ?; Item 6: Same reference criteria used ?; Item 7: Reference criteria independent of Index test ?; Item 8: Repeatability of the Index test ?; Item 9: Repeatability of reference criteria ?; Item 10: Blinding of researchers to reference ?; Item 11: Blinding of researchers to index test ?; Item 12: Availability of clinical data ?; Item 13: Uninterpretable results reported ?; Item 14: Withdrawals explained ?.

Supplementary Table 2: Evaluation of the bias from retrospective cohort studies by the Newcastle-Ottawa Scale (NOS) checklist

	Selection				Comparability	Outcome measurement		
	Representativeness of cases	Controls slection	Exposure definition	Endpoint definition	Comparability of cases and controls on the basis of the design or analysis	Outcome assessment	Follow-up time adequate or not	Follow-up time for cases and controls
Chen 2016 [9]	1	1	1	1	2	0	1	1
Fu 2016 [10]	1	1	1	1	2	1	1	1
Kim 2013 [11]	1	1	1	1	2	1	0	0
Wei 2017 [21]	1	1	1	1	2	0	0	0
Peng 2016 [18]	1	1	1	1	2	0	1	1
Sun 2016 [19]	1	1	1	1	2	0	1	1
Liu 2016 [14]	1	1	1	1	2	0	1	1
Liu 2014 [12]	1	1	1	1	2	0	1	1
Pang 2014 [13]	1	1	1	1	2	0	1	1
Li 2015 [16]	1	1	1	1	2	0	1	1
Zheng 2016 [23]	1	1	1	1	2	1	1	1
Ding 2014 [22]	1	1	1	1	2	0	0	0
Sun 2014 [20]	1	1	1	1	2	0	0	0
Li 2014 [17]	1	1	1	1	2	0	1	1

L'Abbe plot of the diagnostic meta-analysis

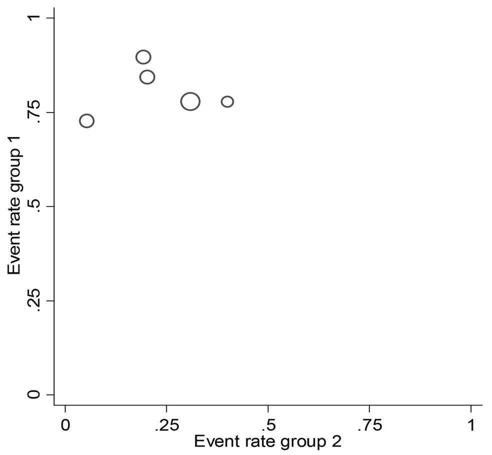

Galbraith plot of the diagnostic meta-analysis

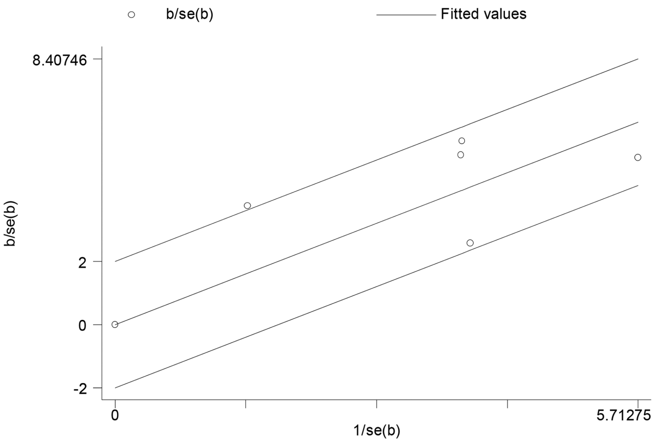

Galbraith plot of the prognostic univariate meta-analysis

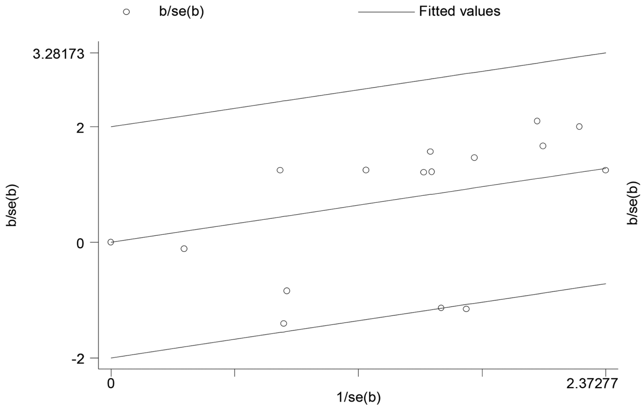

Galbraith plot of the prognostic multivariate meta-analysis

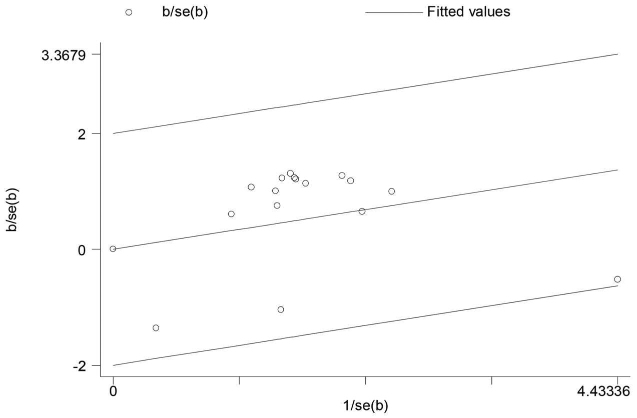

Supplementary Figure 1: Study heterogeneity assessed by visual L'Abbe and Galbraith plots.

Funnel plot of the prognostic univariate meta-analysis

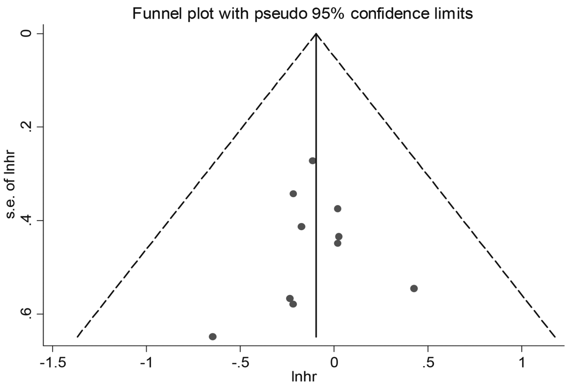

Egger's test of the prognostic univariate meta-analysis

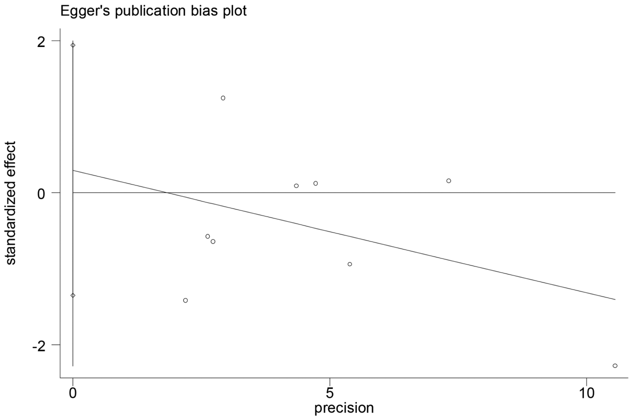

Funnel plot of the prognostic multivariate meta-analysis

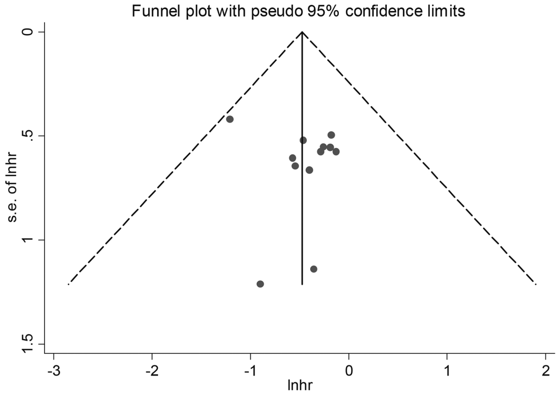

Egger's test of the prognostic multivariate meta-analysis

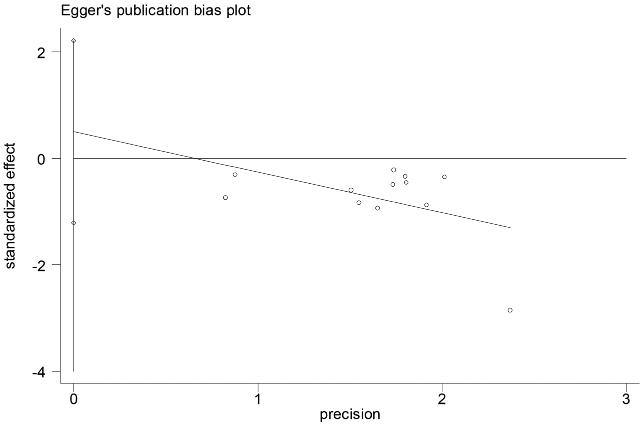

Supplementary Figure 2: Publication bias exmained by the Funnel plot and Egger's test.
